# Data-Driven Model of Postsynaptic Currents Mediated by NMDA or AMPA Receptors in Striatal Neurons

**DOI:** 10.3389/fncom.2022.806086

**Published:** 2022-05-11

**Authors:** Ilaria Carannante, Yvonne Johansson, Gilad Silberberg, Jeanette Hellgren Kotaleski

**Affiliations:** ^1^Science for Life Laboratory, KTH Royal Institute of Technology, Department of Computer Science, Stockholm, Sweden; ^2^Sainsbury Wellcome Centre for Neural Circuits and Behaviour, University College London, London, United Kingdom; ^3^Department of Neuroscience, Karolinska Institutet, Stockholm, Sweden

**Keywords:** decay time constant, double exponential fitting, NMDA receptors, AMPA receptors, postsynaptic current, conductance-based models

## Abstract

The majority of excitatory synapses in the brain uses glutamate as neurotransmitter, and the synaptic transmission is primarily mediated by AMPA and NMDA receptors in postsynaptic neurons. Here, we present data-driven models of the postsynaptic currents of these receptors in excitatory synapses in mouse striatum. It is common to fit two decay time constants to the decay phases of the current profiles but then compute a single weighted mean time constant to describe them. We have shown that this approach does not lead to an improvement in the fitting, and, hence, we present a new model based on the use of both the fast and slow time constants and a numerical calculation of the peak time using Newton's method. Our framework allows for a more accurate description of the current profiles without needing extra data and without overburdening the comptuational costs. The user-friendliness of the method, here implemented in Python, makes it easily applicable to other data sets.

## 1. Introduction

Neurons communicate with each other *via* synapses which can be distinguished on the basis of their mechanism of transmission in electrical and chemical synapses. The synaptic transmission is a highly dynamic process. Because of the importance and abundance of synapses it is extremely useful to have a computationally accurate and efficient framework to simulate them. In electrical synapses, the signal in the form of current flows directly from one neuron to another through gap junctions. In contrast, chemical synapses enable neurons communication through neurotransmitters which are released from the presynaptic neuron and are received by neurotransmitter receptors on the postsynaptic neuron. The main excitatory neurotransmitter in the brain is glutamate which co-activates (among others) postsynaptic ionotropic NMDA and AMPA receptors.

Here, we focus on modeling the postsynaptic current (PSC) of these receptors using the conductance-based models. In particular: AMPA synaptic currents are computed as *I*_*AMPA*_(*t*) = *g*(*t*)(*V*(*t*) − *E*_*rev*_), where *g* is the conductance, *V* the membrane potential and *E*_*rev*_ the reversal potential; while NMDA synaptic currents depend also on the *Mg*^2+^ block and hence are estimated as *I*_*NMDA*_(*t*) = *g*(*t*)*Mg*(*V*(*t*))(*V*(*t*) − *E*_*rev*_) (see Methods).

A common function used to describe synaptic conductance profiles *g*(*t*), following activation, is a double exponential, where one exponential describes the rising phase and the other the decay phase of the PSC. Consequently two time constants are set independently, τ_*rise*_ and τ_*decay*_. This formulation allows two expressions with which the peak time and the normalization factor for the amplitude are calculated. However, the decay phase of the PSC is not always well described using a single exponential, so in many cases a double exponential is used to fit the decay phase. Nevertheless, often one weighted mean time constant (τ_*w*_) is extracted and hence a single exponential function, with such time constant, is used to model the decay (Stocca and Vicini, 1998; Chapman et al., 2003).

Here, we show that there is not much improvement in using the weighted mean time constant compared to the single decay time constant, and for this reason we present a new approach based on the use of both (the fast and slow) time constants (τ_*f*_ and τ_*s*_).

Hence, the synaptic conductance profiles are described using three exponentials, one for the rising and two for the decay phases. In this case no closed-form expression exists for the peak time, and it is calculated numerically using Newton's method. Here, we apply this new approach to describe the postsynaptic currents of NMDA and AMPA receptors expressed by different types of striatal neurons in the mouse, and we show that this model describes the synaptic responses of striatal neurons more accurately than the standard methods. The implementation is done in Python and the simulations in NEURON (Carnevale and Hines, 2006). The framework is general and can be applicable to other data sets, for example to describe the postsynaptic currents in other brain regions.

The raw data underlying this model was acquired *ex vivo* by obtaining whole-cell patch clamp recordings of striatal neurons while activating different striatal inputs, namely primary motor cortex (M1), primary somatosensory cortex (S1), and parafascicular nucleus (PF), using optogenetic stimulation.

## 2. Materials and Methods

### 2.1. Data Acquisition

All animal procedures were performed in accordance with the national guidelines and approved by the local ethics committee of Stockholm, Stockholms Norra djurförsöksetiska nämnd, under an ethical permit to G. S. (N12/15). D1-Cre and D2-Cre (EY262 and ER44 line, GENSAT), SOM-Cre, PV-Cre, and ChAT-Cre mice were crossed with tdTomato reporter mice (stock #018973, #017320, #006410, and #007909, the Jackson laboratory). Viral injections and whole-cell patch clamp recordings were performed as described previously (Johansson and Silberberg, 2020).

In brief, mice were injected with virus (AAV2-CamKIIa-eYFP-ChR2, #26969, addgene) in M1, S1, or PF. Three to nine weeks later, brain slices (250μm) were prepared in a cutting buffer solution containing 205 mM sucrose, 10 mM glucose, 25 mM NaHCO_3_, 2.5 mM KCl, 1.25 mM NaH_2_PO_4_, 0.5 mM CaCl_2_, and 7.5 mM MgCl_2_ before being kept for 30–60 min at 35°*C* in a submerged chamber filled with artificial cerebrospinal fluid (ACSF) saturated with 95% oxygen and 5% carbon dioxide. ACSF was composed of 125 mM NaCl, 25 mM glucose, 25 mM NaHCO_3_, 2.5 mM KCl, 2 mM CaCl_2_, 1.25 mM NaH_2_PO_4_, and 1 mM MgCl_2_. Neurons were visualized using infrared differential interference contrast microscopy or epifluorescence (Zeiss FS Axioskop, Oberkochen, Germany; X-cite, 120Q, Lumen Dynamics). Whole-cell patch clamp recordings were acquired in oxygenated ACSF at 35°*C* with borosilicate pipettes. Voltage-clamp recordings were obtained with a caesium-based intracellular composed of 100 mM CsMeSO_3_, 10 mM CsCl, 10 mM HEPES, 4 mM Mg-ATP, 0.3 mM Na-GTP, 10 mM Na_2_-phosphocreatine, and 10 mM tetraethylammonium chloride (TEA-Cl), and pipette resistances of 3−5 MOhm. Postsynaptic currents were measured at a clamping potential of −70 and +40 mV to estimate the NMDA to AMPA ratio. Current-clamp recordings were acquired with an intracellular solution consisting of 130 mM K-gluconate, 5 mM KCl, 10 mM HEPES, 4 mM Mg-ATP, 0.3 mM GTP, 10 mM Na2-phosphocreatine (pH 7.25, osmolarity 285 mOsm) and pipette resistances between 6–8 MOhm. Recordings were amplified using a MultiClamp 700B amplifier (Molecular Devices, CA, USA), filtered at 2 kHz, digitized at 10–20 kHz using ITC-18 (HEKA Elektronik, Instrutech, NY, USA), and acquired using custom-made routines running on Igor Pro (Wavemetrics, OR, USA). Throughout all recordings pipette capacitance and access resistance were compensated for and data were discarded when access resistance increased beyond 30 MOhm. All recordings were acquired in the presence of gabazine (GBZ) (10 μM) and in a subset of experiments APV (50 μM) was additionally applied. Drugs were bath-applied and washed in for at least 7.5 min before acquiring data. Optogenetic stimulation (wavelength 465 nm) was delivered through the 64x objective lens. 2 ms light pulses were used for activating cortical or thalamic terminals in dorsal striatum. All current- and voltage-clamp recordings obtained in GBZ alone have been previously published (Johansson and Silberberg, 2020).

### 2.2. Data Analysis

M1, S1, and PF were optogenetically activated and whole-cell patch clamp recordings of striatal projection neurons (dSPN and iSPN), fast-spiking (FS), low-threshold spiking (LTS), and cholinergic (ChIN) interneurons were acquired. In some FS cells, the recorded NMDA current traces peaked before or at the same time as the AMPA current traces. Since NMDA type receptors typically possess slower kinetics than AMPA type receptors (Myme et al., 2003, and since it has been shown that not all FS express NMDA receptors (Nyiri et al., 2003; Matta et al., 2013) those traces were excluded (see Supplementary Figure 1). In Table 1 all the data used is collected and the average traces are plotted in Supplementary Figure 2. The current peak at −70 mV was extracted as the AMPA component while the NMDA current was quantified as the average current 50–60 ms after the stimulation at +40 mV (see Supplementary Figure 3). The ratio between these values was calculated recording by recording (cell by cell), and then for each input region and cell type the average of these values was estimated as the NMDA to AMPA ratio. To fit the (rise and decay) time constants, synaptic responses were recorded at two different voltages (−70 and +40 mV) first in the presence of GBZ followed by APV bath application. In particular, the AMPA component was subtracted from the raw trace recorded at +40 mV in the presence of GBZ (see Supplementary Figure 4). This allowed to achieve a pharmacological separation between the AMPA and NMDA components. For the input regions and cell types where these recordings (in two different bath applications) were not available, simulations were used to estimate the NMDA currents (see Supplementary Figure 5).

**Table 1:** Input region and target cell.

		**Cell type**				
		**dSPN**	**iSPN**	**FS**	**ChIN**	**LTS**
Input region	M1-contra	12	11	0/8*	-	-
	M1-ipsi	14	14	3/12*	4	5
	S1	15	15, 1	2/5*	3	-
	PF	8, 1	21, 2	0/7*	9, 1	-

*For each input region and target cell type the number of traces used is reported. Some fast-spiking cell (FS) traces were excluded because they showed no or negligible NMDA currents. In particular no NMDA responses were observed in FS when activating PF or contralateral M1. When recordings were not available the corresponding space is left blank (-). Numbers in blue represent the additional traces for which the recordings in two different bath applications (GBZ and APV) were available*.

### 2.3. Model

Generally synaptic conductances are modeled with the following double exponential kinetics for *t* ≥ *t*_0_:


(1) $$\begin{array}{r} g_{syn}\left(t\right)=ḡ\cdot K\cdot \left(e^{-\left(t-t_{0}\right)/\tau_{decay}}-e^{-\left(t-t_{0}\right)/\tau_{rise}}\right), \end{array}$$

where ḡ is the peak synaptic conductance, *K* is the normalization factor, *t*_0_ is the time of the presynaptic spike, and τ_*rise*_ and τ_*decay*_ are the PSC rise and decay time constant respectively. The peak time, i.e., the time at which (1) reaches the maximum, is found imposing the derivative of (1) equal to zero and corresponds to:


(2) $$\begin{array}{r} t_{peak}=t_{0}+\frac{\tau_{decay}\cdot \tau_{rise}}{\tau_{decay}-\tau_{rise}}\cdot \ln\left(\frac{\tau_{decay}}{\tau_{rise}}\right). \end{array}$$

The normalization factor is a constant such that (1) evaluated at *t*_*peak*_ equals ḡ and corresponds to:


(3) $$\begin{array}{r} K=\frac{1}{e^{-\left(t_{peak}-t_{0}\right)/\tau_{decay}}-e^{-\left(t_{peak}-t_{0}\right)/\tau_{rise}}}. \end{array}$$

In order to obtain a better fit of PSC we propose to approximate the decay phase using two exponentials. It is indeed a very common practice in numerical simulations to use a double exponential fitting for the decay


(4) $$\begin{array}{r} I_{f}e^{-\left(t-t_{0}\right)/\tau_{f}}+I_{s}e^{-\left(t-t_{0}\right)/\tau_{s}}, \end{array}$$

but then compute a weighted mean time constant τ_*w*_ as follows (Stocca and Vicini, 1998


(5) $$\begin{array}{r} \tau_{w}=\frac{I_{f}}{I_{f}+I_{s}}\tau_{f}+\frac{I_{s}}{I_{f}+I_{s}}\tau_{s}, \end{array}$$

and use it as τ_*decay*_ in the model. Although potentially numerically more efficient (than using both τ_*rise*_ and τ_*decay*_), τ_*w*_ is generally not even better than the τ_*decay*_ obtained using the single exponential (see Results and Discussion).

Here, we also use a double exponential function to fit the decay but both time constants (τ_*f*_ and τ_*s*_) and both coefficients (*I*_*f*_ and *I*_*s*_) are used to describe it. In this case, the equation which models the synaptic conductance can be written as:


(6) $$\begin{array}{r} g_{syn}\left(t\right)=ḡ\cdot K\cdot \left(I_{f}e^{-\left(t-t_{0}\right)/\tau_{f}}+I_{s}e^{-\left(t-t_{0}\right)/\tau_{s}}-\tilde{K}e^{-\left(t-t_{0}\right)/\tau_{r}}\right), \end{array}$$

where $\tilde{K,}$ corresponding to the sum of *I*_*f*_ and *I*_*s*_, is included to ensure that Equation (6) evaluated at *t*_0_ gives zero. Hence, in order to find the time of peak *t*_*peak*_, the derivative of Equation (6) has to be set equal to zero. Doing so, the following equation is obtained


(7) $$\begin{array}{r} g_{syn}^{\prime }\left(t\right)=ḡ\cdot K\cdot \left(-\frac{I_{f}}{\tau_{f}}e^{-\left(t-t_{0}\right)/\tau_{f}}-\frac{I_{s}}{\tau_{s}}e^{-\left(t-t_{0}\right)/\tau_{s}}+\frac{\tilde{K}}{\tau_{r}}e^{-\left(t-t_{0}\right)/\tau_{r}}\right)=0. \end{array}$$

From Equation (7), we find the following equation to which *t*_*peak*_ is solution:


(8) $$\begin{array}{r} \frac{I_{f}}{\tau_{f}}e^{-\left(t-t_{0}\right)/\tau_{f}}+\frac{I_{s}}{\tau_{s}}e^{-\left(t-t_{0}\right)/\tau_{s}}=\frac{\tilde{K}}{\tau_{r}}e^{-\left(t-t_{0}\right)/\tau_{r}}. \end{array}$$

It is possible to simplify this expression by taking the logarithm of both sides:


(9) $$\begin{array}{r} \log\left(\frac{I_{f}}{\tau_{f}}e^{-\left(t-t_{0}\right)/\tau_{f}}+\frac{I_{s}}{\tau_{s}}e^{-\left(t-t_{0}\right)/\tau_{s}}\right)=\log\frac{\tilde{K}}{\tau_{r}}-\frac{t-t_{0}}{\tau_{r}}. \end{array}$$

In the end, we arrive at the expression:


(10) $$\begin{array}{r} F\left(t\right):=t-\frac{\tau_{r}\tau_{f}}{\tau_{r}-\tau_{f}}\log\left(\frac{I_{f}}{\tilde{K}}\frac{\tau_{r}}{\tau_{f}}+\frac{I_{s}}{\tilde{K}}\frac{\tau_{r}}{\tau_{s}}e^{\frac{\left(t-t_{0}\right)\left(\tau_{s}-\tau_{f}\right)}{\tau_{f}\tau_{s}}}\right)-t_{0}=0. \end{array}$$

As we said, *t*_*peak*_ is solution to this equation, and to calculate it numerically, we can use Newton's method on the function *F*. In order to use Newton's method on *F*, we also need its derivative:


(11) $$\begin{array}{r} F^{\prime }\left(t\right)=1-\frac{I_{s}\tau_{r}\tau_{f}\left(\tau_{s}-\tau_{f}\right)}{\tau_{s}\left(\tau_{r}-\tau_{f}\right)}{\left(I_{f}\tau_{s}+I_{s}\tau_{f}e^{\frac{\left(t-t_{0}\right)\left(\tau_{s}-\tau_{f}\right)}{\tau_{f}\tau_{s}}}\right)}^{{\;}^{-1}}e^{\frac{\left(t-t_{0}\right)\left(\tau_{s}-\tau_{f}\right)}{\tau_{f}\tau_{s}}}. \end{array}$$

Newton's method is very convenient for this setting because of its properties: quadratic convergence which implies that very few steps are necessary for a result accurate up to machine precision (< 6); local convergence which means that if we give a good starting guess, we will always find the result; ease of implementation, given the simplicity of the method.

A Python version of the method is showed in Algorithm 1.

**Algorithm 1:** A Python version of Newton's method for a generic function f = *F* and its derivative fp = *F*′ to find the point t^*^such that *f*(t^*^) = 0. In our case *F* and *F*′depend on the fitted parameters τ_*r*_, τ_*f*_, τ_*s*_, *I*_*f*_, and *I*_*s*_.

def Newton(t_in, f, fp, TOL, MaxIter)
t = t_in;
d = 1;
niter = 1;
while abs(d)>TOL && niter < =MaxIter:
d = f(t)/fp(t)
t -= d
niter += 1
return t

The normalization factor *K*, as before, is a constant such that the equation describing the synaptic conductance evaluated in *t*_*peak*_ equals ḡ. When the synaptic conductance is described using Equation (1) it corresponds to Equation (3), while when the synaptic conductance is Equation (6), the normalization factor is:


(12) $$\begin{array}{r} K=\frac{1}{I_{f}e^{-\left(t_{peak}-t_{0}\right)/\tau_{f}}+I_{s}e^{-\left(t_{peak}-t_{0}\right)/\tau_{s}}-\tilde{K}e^{-\left(t_{peak}-t_{0}\right)/\tau_{rise}}}. \end{array}$$

AMPA synaptic currents are then computed as:


(13) $$\begin{array}{r} I_{syn}\left(t\right)=g_{syn}\left(t\right)\left(V\left(t\right)-E_{syn}\right). \end{array}$$

NMDA currents, depending also on the magnesium block:


(14) $$\begin{array}{r} {Mg}_{syn}\left(V\right)=\frac{1}{1+e^{-aV}\left(\left[Mg^{2+}/b\right)\right]}, \end{array}$$

where [*Mg*^2+^] is the extracellular magnesium concentration and *a* and *b* are constants (Jahr and Stevens, 1990), are computed as:


(15) $$\begin{array}{r} I_{syn}\left(t\right)=g_{syn}\left(t\right){Mg}_{syn}\left(V\left(t\right)\right)\left(V\left(t\right)-E_{syn}\right). \end{array}$$

The Python library SciPy is used to find the optimal set of parameters that best fits the average traces. Python codes for the fitting procedure and the Newton's method implementation are available at github.com/IlaCar/PSC_double_decay_fitting.

### 2.4. NMODL Implementation

In order to simulate in NEURON the postsynaptic conductance and current of NMDA and AMPA receptors we updated the mod file available here (tmglut.mod, ModelDB https://senselab.med.yale.edu/ModelDB/, accession number 237653). The main changes we had to make are: the creation of two new states C_ampa and C_nmda which account for the second decay time constant, the modification of how tp_ampa, tp_nmda, factor_ampa, and factor_nmda are computed, where in particular the first two are the output of the Newton's method. We also apply a correction to the original (Jahr and Stevens, 1990) constants *a* and *b* based on the observations in Ecker et al. (2020). Also these files are available in the same repository.

## 3. Results

**Figure 1:**
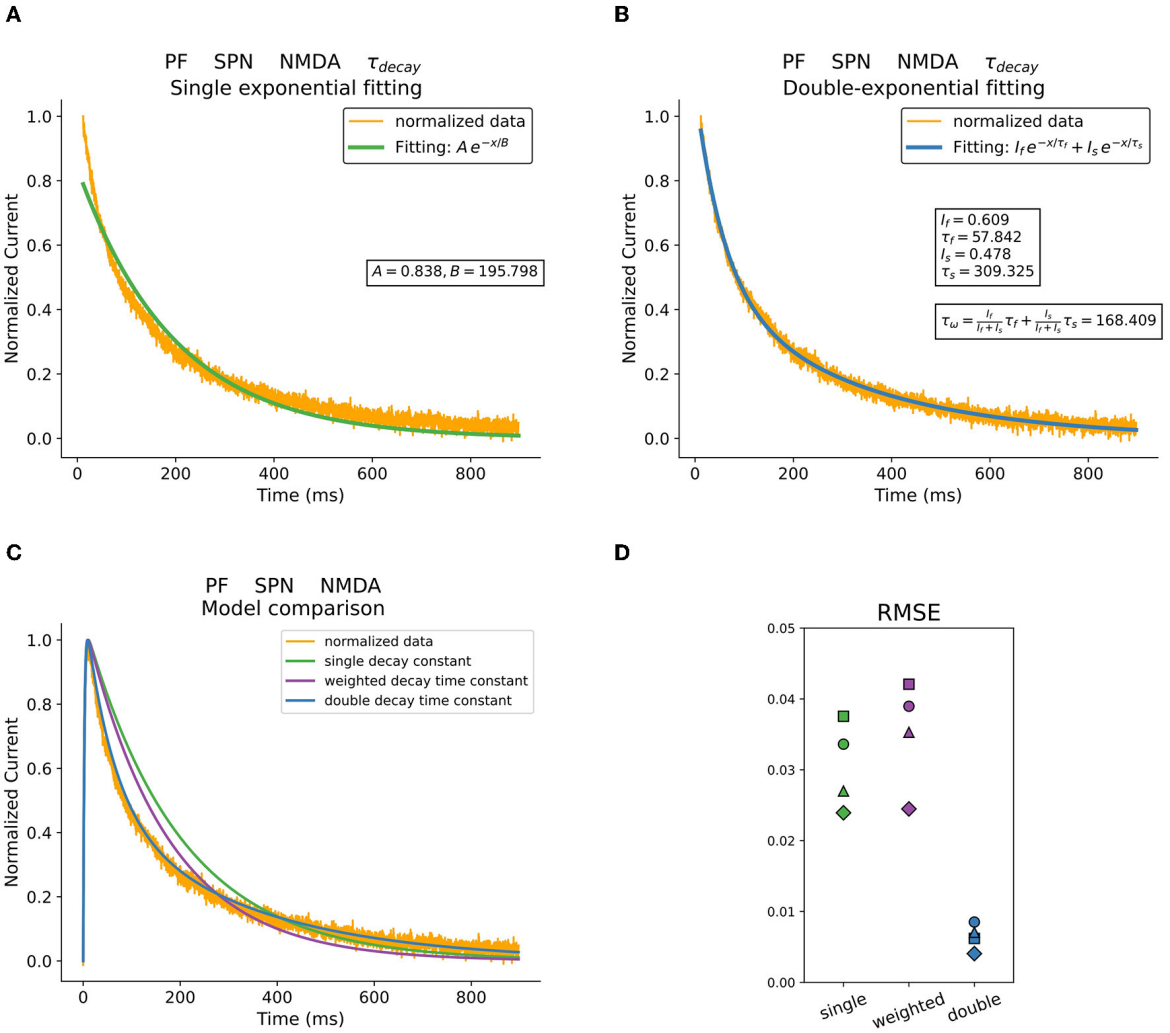
Decay fitting procedures and resulting models. **(A)** Mono exponential decay time fitting. **(B)** Double exponential decay time fitting and estimated weighted time constant. **(C)** Comparison between models obtained using the different fitting procedures and parameters. Original data is shown in orange. **(D)** RMSE of three different fitting methods describing postsynaptic currents in striatal neurons when stimulating PF. Data was acquired in voltage-clamp at +40 mV and in the presence of GBZ and APV.

Postsynaptic currents were obtained in whole-cell recordings from striatal neurons while activating corticostriatal or thalamostriatal terminals with brief light pulses (see Materials and Methods). The decay phase of synaptic responses was fitted using single, weighted, or double exponential decay time constants. An example of the different decay time constant fitting procedures previously described is shown in Figure 1. In particular, the NMDA current trace recorded in an SPN when photostimulating thalamostriatal terminals is represented. Figures 1A,B illustrate a mono exponential and a double exponential fitting, respectively. To study the behavior of the weighted mean time constant τ_*w*_, it was extracted using (5). The models resulting from these fittings are shown in Figure 1C.

The root-mean-square error (RMSE) was calculated to compare these fitting procedures and the results are presented in Figure 1D. Specifically, the RMSE of the different fitting procedures are plotted for the NMDA currents recorded in response to PF stimulation. The corresponding example regarding AMPA currents is shown in Supplementary Figure 6. The double exponential fitting procedure performs up to several times better that the others, especially when describing the slower kinetics of the NMDA currents.

**Figure 2:**
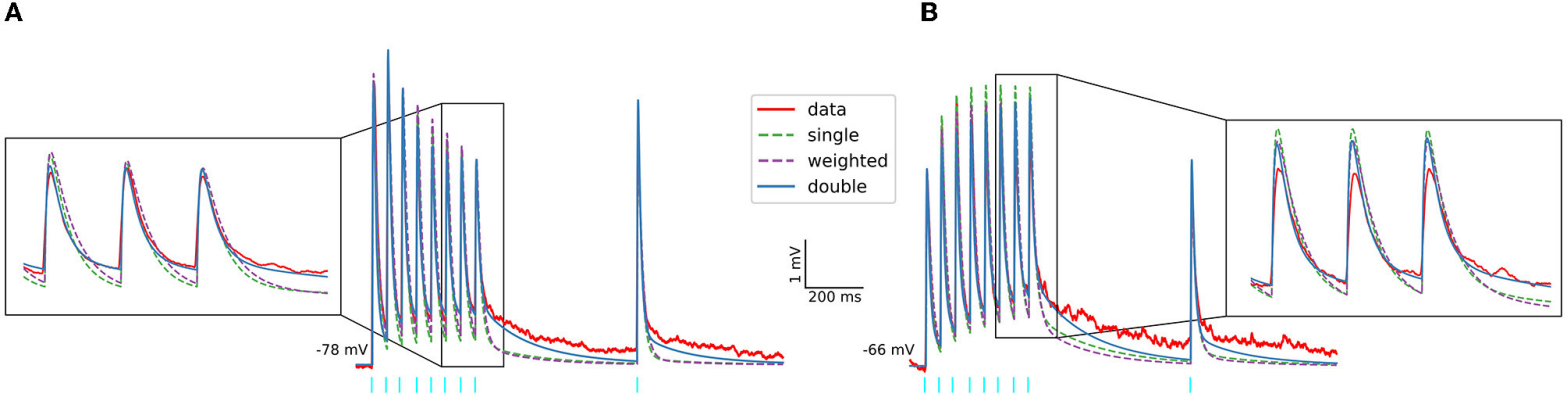
Experimental and *in-silico* excitatory postsynaptic potentials of SPNs evoked by optogenetic activation of PF. Protocol includes 8 pulses at 20 Hz followed by a recovery pulse (light blue bars). Two different experimental EPSP are shown. In **(A)** the glutamatergic synapse models used for the simulation (in NEURON+Python) are based on the NMDA and AMPA currents which were pharmacologically separated, while the glutamatergic synapses models used in **(B)** are based on the estimated NMDA currents.

Moreover, we implemented the different postsynaptic current models for each input region and neuron type (see Materials and Methods) and used them to describe the dynamics of the corticostriatal and thalamostriatal glutamatergic synapses following the same procedure as in (Hjorth et al., 2020). An example of the results, including two different experimental traces and their *in silico* simulations using NEURON+Python, is shown in Figure 2. In Figure 2A, the glutamatergic synapse model is based on the NMDA and AMPA currents which were pharmacologically separated, while in Figure 2B the NMDA current was estimated with the procedure described in Supplementary Figure 5. In both scenarios, the excitatory postsynaptic potentials (EPSPs) obtained in response to 20 Hz stimulation (red lines) are better described when using the double exponential decay for AMPA and NMDA currents models (blue lines).

One possible disadvantage of our method could have been the increase of computational time necessary to simulate the synapses. We timed the performance of the models when simultaneously stimulating up to three thousand glutamatergic synapses described using the Tsodyks-Markram model (Uziel et al., 2000) and distributed on a multicompartmental cell. The simulations done using glutamatergic synapse models based on the AMPA and NMDA models of postsynaptic current presented here were in the heaviest scenario (three thousand simultaneously active synapses on a single cell) only around 10% slower than the ones using glutamatergic synapse models obtained using the weighted time constant (or the mono exponential time constant). Hence, our method presents a good balance between computational efficacy and accuracy.

## 4. Discussion

We focused on modeling the postsynaptic current (PSC) of NMDA and AMPA receptors using conductance-based models. The decay phase is usually modeled using a single exponential function, but sometimes the decay time constant can result from a double exponential fitting procedure, which parameters are combined (in a sort of weighted mean) to obtain one decay time constant. In contrast, our method uses all the parameters that result from the double exponential fitting and improves considerably the description of the PSC (up to 10 times). The absence of a formula to calculate the peak time (available for the classical approaches) is easily and efficiently bypassed by using Newton's method. Our method allows for a more effective use of the available data, especially to model the slower kinetics of the NMDA currents, and this can be crucial when describing dendritic nonlinearities (Plotkin et al., 2011; Du et al., 2017; Lindroos and Hellgren Kotaleski, 2021).

The drawback is a slowdown of the performance (up to 10% when simulating three thousand simultaneously active synapses on a single neuron), but considering the improvement of description of the currents and the increasing availability of high performance computers, we believe it is an acceptable drawback.

Simulations of large-scale detailed data-driven neural networks are a powerful approach to understand brain functionalities and, as a consequence, different microcircuits have been reconstructed *in silico* (Markram et al., 2015; Billeh et al., 2020; Hjorth et al., 2020). We are currently working on the integration of the NMDA and AMPA models, presented here, into the striatal large scale network. Our workflow is general and applicable to potentially describe all types of synaptic currents in any region of the brain.

## Data Availability Statement

The datasets for this study and the implemented codes can be found at: https://github.com/IlaCar/PSC_double_decay_fitting. Further inquiries can be directed to the corresponding authors.

## Author Contributions

IC conceptualized the study, performed calculations, derived the models, and analysed the results. YJ performed the experiments and provided experimental data sets. GS supervised the experiments. IC and YJ analysed data. IC, YJ, GS, and JK wrote the manuscript. All authors contributed to the article and approved the submitted version.

## Funding

The study was supported by the Swedish Research Council (VR-M-2017-02806 and VR-M-2020-01652), Swedish e-Science Research Centre (SeRC), EU/Horizon 2020 no. 945539 (HBP SGA3), KTH Digital Futures to JK, Wallenberg Academy Fellow prolongation (KAW 2017.0273), Hjärnfonden (FO2021-0333), VR-M (2019-01254) to GS, and international VR postdoc grant to YJ, registration number 2020-06365.

## Conflict of Interest

The authors declare that the research was conducted in the absence of any commercial or financial relationships that could be construed as a potential conflict of interest.

## Publisher's Note

All claims expressed in this article are solely those of the authors and do not necessarily represent those of their affiliated organizations, or those of the publisher, the editors and the reviewers. Any product that may be evaluated in this article, or claim that may be made by its manufacturer, is not guaranteed or endorsed by the publisher.

## References

[B1] Billeh Y. N. Cai B. Gratiy S. L. Dai K. Iyer R. Gouwens N. W. . (2020). Systematic integration of structural and functional data into multi-scale models of mouse primary visual cortex. Neuron 106, 388–403. 10.1016/j.neuron.2020.01.040 32142648

[B2] Carnevale N. T. Hines M. L. (2006). The Neuron Book. Cambridge: Cambridge University Press.

[B3] Chapman D. E. Keefe K. A. Wilcox K. S. (2003). Evidence for functionally distinct synaptic nmda receptors in ventromedial versus dorsolateral striatum. J. Neurophysiol. 89, 69–80. 10.1152/jn.00342.2002 12522160

[B4] Du K. Wu Y.-W. Lindroos R. Liu Y. Rózsa B. Katona G. . (2017). Cell-type–specific inhibition of the dendritic plateau potential in striatal spiny projection neurons. Proc. Natl. Acad. Sci. 114, E7612–E7621. 10.1073/pnas.1704893114 28827326 PMC5594658

[B5] Ecker A. Romani A. Sáray S. Káli S. Migliore M. Falck J. . (2020). Data-driven integration of hippocampal ca1 synaptic physiology in silico. Hippocampus 30, 1129–1145. 10.1002/hipo.23220 32520422 PMC7687201

[B6] Hjorth J. J. Kozlov A. Carannante I. Nylén J. F. Lindroos R. Johansson Y. . (2020). The microcircuits of striatum in silico. Proc. Natl. Acad. Sci. U.S.A. 117, 9554–9565. 10.1073/pnas.2000671117 32321828 PMC7197017

[B7] Jahr C. E. Stevens C. F. (1990). Voltage dependence of nmda-activated macroscopic conductances predicted by single-channel kinetics. J. Neurosci. 10, 3178–3182. 1697902 10.1523/JNEUROSCI.10-09-03178.1990 PMC6570236

[B8] Johansson Y. Silberberg G. (2020). The functional organization of cortical and thalamic inputs onto five types of striatal neurons is determined by source and target cell identities. Cell Rep. 30, 1178–1194. 10.1016/j.celrep.2019.12.095 31995757 PMC6990404

[B9] Lindroos R. Hellgren Kotaleski J. (2021). Predicting complex spikes in striatal projection neurons of the direct pathway following neuromodulation by acetylcholine and dopamine. Eur. J. Neurosci. 53, 2117–2134. 10.1111/ejn.14891 32609903

[B10] Markram H. Muller E. Ramaswamy S. Reimann M. W. Abdellah M. Sanchez C. A. . (2015). Reconstruction and simulation of neocortical microcircuitry. Cell 163, 456–492. 10.1016/j.cell.2015.09.029 26451489

[B11] Matta J. A. Pelkey K. A. Craig M. T. Chittajallu R. Jeffries B. W. McBain C. J. (2013). Developmental origin dictates interneuron ampa and nmda receptor subunit composition and plasticity. Nat. Neurosci. 16, 1032–1041. 10.1038/nn.3459 23852113 PMC4132661

[B12] Myme C. I. Sugino K. Turrigiano G. G. Nelson S. B. (2003). The nmda-to-ampa ratio at synapses onto layer 2/3 pyramidal neurons is conserved across prefrontal and visual cortices. J. Neurophysiol. 90, 771–779. 10.1152/jn.00070.2003 12672778

[B13] Nyiri G. Stephenson F. Freund T. Somogyi P. (2003). Large variability in synaptic n-methyl-d-aspartate receptor density on interneurons and a comparison with pyramidal-cell spines in the rat hippocampus. Neuroscience 119, 347–363. 10.1016/s0306-4522(03)00157-x 12770551

[B14] Plotkin J. L. Day M. Surmeier D. J. (2011). Synaptically driven state transitions in distal dendrites of striatal spiny neurons. Nat. Neurosci. 14, 881–888. 10.1038/nn.2848 21666674 PMC3235762

[B15] Stocca G. Vicini S. (1998). Increased contribution of nr2a subunit to synaptic nmda receptors in developing rat cortical neurons. J. Physiol. 507, 13–24. 9490809 10.1111/j.1469-7793.1998.013bu.x PMC2230772

[B16] Uziel A. Tsodyks M. Markram H. (2000). Synchrony generation in recurrent networks with frequency-dependent synapses. J. Neurosci. 20, RC50. 10.1523/JNEUROSCI.20-01-j0003.2000 10627627 PMC6774142

