# Supplementary material for: Data-Driven Model of Postsynaptic Currents Mediated by NMDA or AMPA Receptors in Striatal Neurons
Source: Front Comput Neurosci. 2022 May 11;16:806086. doi: 10.3389/fncom.2022.806086 (PMC9130461; doi:10.3389/fncom.2022.806086)
Supplement: Supplementary file 1 [file Data_Sheet_1.pdf]

Supplementary Material

1 SUPPLEMENTARY TABLES AND FIGURES

1.1 Figures

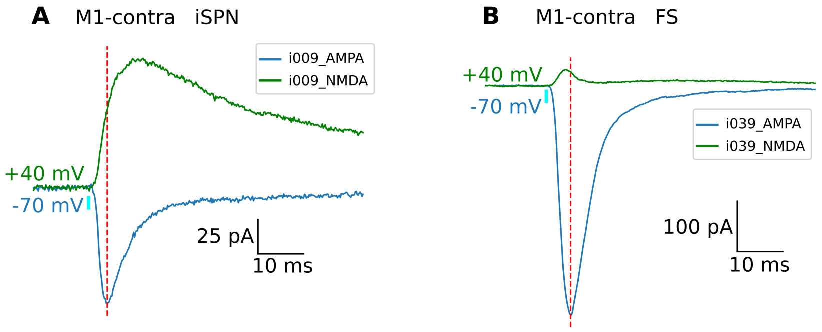

Figure S1. Comparison between valid and excluded NMDA traces. **(A)** Example of AMPA and NMDA currents in an iSPN recorded at -70 and $+40$ mV, respectively. **(B)** Example of AMPA and NMDA current traces recorded in an FS interneuron. The current recorded at $+40$ mV peaks before the AMPA current, suggesting stimulation of M1 inputs did not activate NMDA receptors in this FS. The vertical dotted line represents the time at which the AMPA current peaks and the light blue line represents the optogenetic photostimulation.

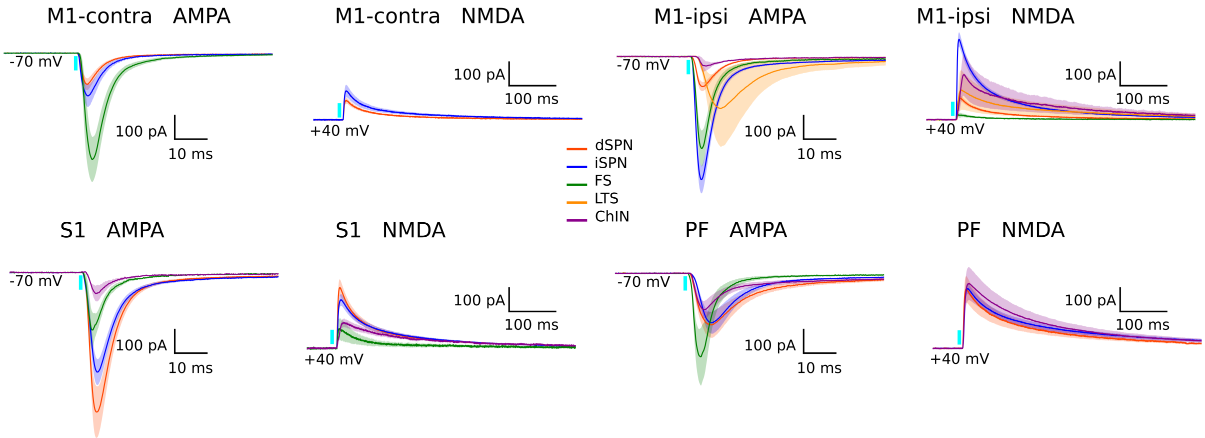

Figure S2. Representative average of AMPA and NMDA currents recprded in the presence of gabazine. The recordings were obtained in striatal neurons by activating M1 (top), S1 (bottom left) and PF (bottom right). Shaded regions represent the SEM.

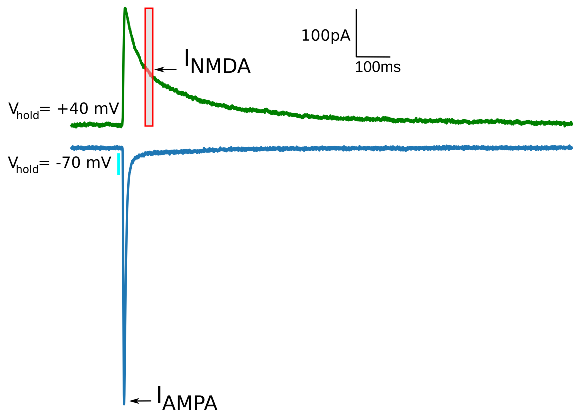

Figure S3. Extraction of the NMDA to AMPA ratio. The current peak at -70 mV was extracted as the AMPA component while the average value between 50-60 ms after the light stimulation at $+40$ mV as the NMDA component.

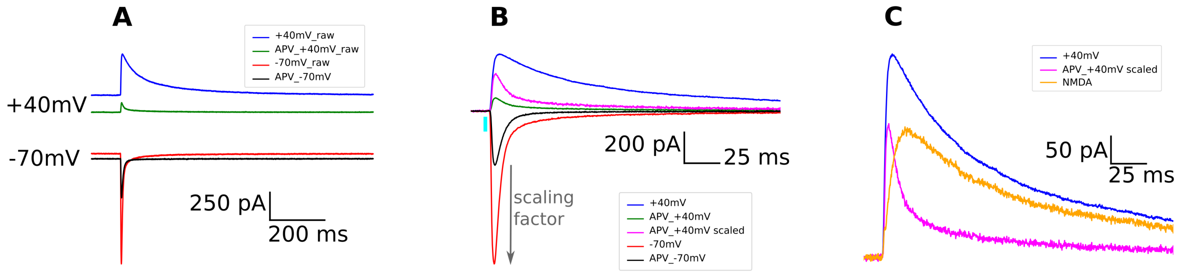

Figure S4. NMDA current isolation. The cell was recorded at two different voltages (-70 and $+40$ mV) in BGZ, before and after additional bath-application of APV. In (A) the raw traces are plotted. Since APV was bath-applied for at least 7.5 minutes before acquiring the next recordings, the amplitude of the new AMPA component was scaled (B). The outward ($+40$ mV) currents recorded after the application of APV were scaled according to the AMPA amplitudes obtained at -70 mV. In (C) the AMPA component (magenta trace) was subtracted from the $+40$mV trace (blue trace) leading to the new estimated NMDA trace (orange trace).

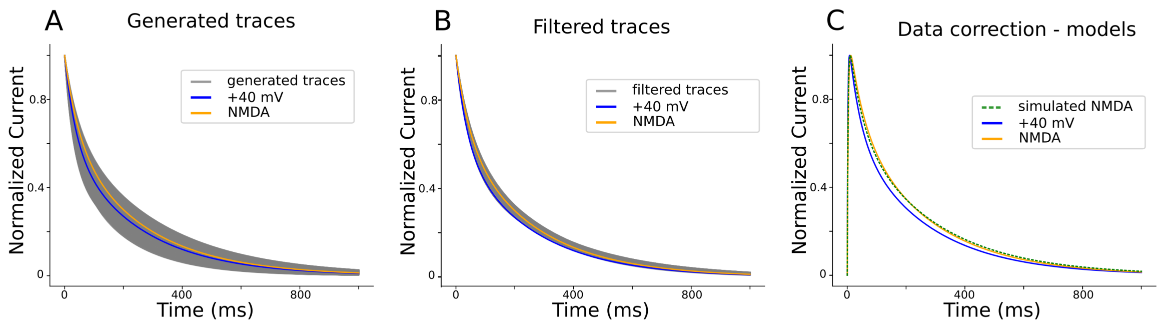

Figure S5. Simulated NMDA traces. When the AMPA component could not be directly subtracted from the current recorded at $+40$ mv (APV traces not available), data-driven simulations were used. In particular, we observed that the fitting parameters of the current traces recorded at $+40$ mV (blue) and those recorded with APV (corrected NMDA currents, yellow) vary within a 20% interval. Moreover, the weighted time decay of the NMDA current increases between 5% and 19%. In (A) a thousand double exponential curves (gray) were randomly generated such that their parameters (I_s, τ_s, I_f, τ_f) are within a 20% interval of the corresponding parameters defining the control trace recorded at $+40$ mV. These traces were then filtered such that their weighted time decay was between 5% and 19% slower than the weighted time decay of the control trace. Finally, curves slower than the control trace were excluded. These filtering criteria led to the remaining traces in (B). In (C) the average of the filtered traces gives the simulated NMDA trace (green). The similarity between the simulated NMDA trace and the pharmacologically isolated NMDA trace can be appreciated.

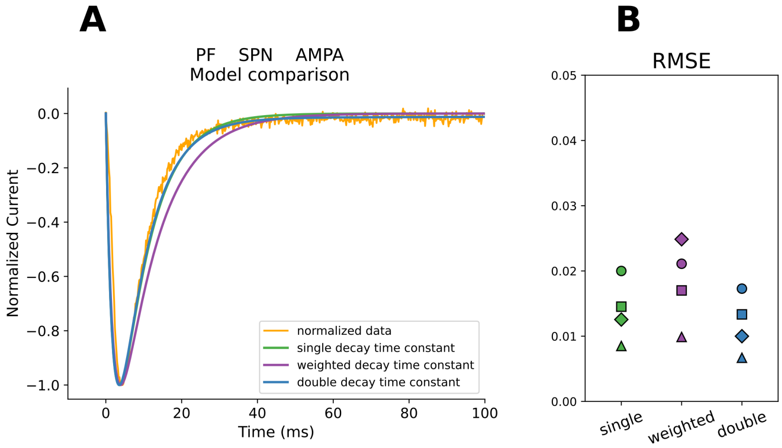

Figure S6. Comparison between AMPA models obtained using the different fitting procedures and parameters (A). Original data is shown in orange. RMSE of the three different fitting methods describing postsynaptic currents in striatal neurons when stimulating PF (B). Data was acquired in voltage-camp at -70 mV.
